# *Clostridium tyrobutyricum* Protects against LPS-Induced Colonic Inflammation via IL-22 Signaling in Mice

**DOI:** 10.3390/nu13010215

**Published:** 2021-01-13

**Authors:** Zhiping Xiao, Lujie Liu, Yuyue Jin, Xun Pei, Wanjing Sun, Minqi Wang

**Affiliations:** The Key Laboratory of Molecular Animal Nutrition, Ministry of Education, College of Animal Sciences, Zhejiang University, Hangzhou 310058, China; xiaozp@zju.edu.cn (Z.X.); liulj@zju.edu.cn (L.L.); jinyuyue@zju.edu.cn (Y.J.); pxpeixun@zju.edu.cn (X.P.); sunwanjing@zju.edu.cn (W.S.)

**Keywords:** *C. tyrobutyricum*, colon, inflammation, immune cells, IL-22

## Abstract

This study aimed to investigate the effects of *Clostridium tyrobutyricum (C. tyrobutyricum)* on colonic immunity and the role of IL-22 in the protective function of *C. tyrobutyricum*. Mice were supplemented with 10^8^ CFU/mL *C. tyrobutyricum* daily for 20 days, followed by injecting with LPS for 24 h. In vivo interference of IL-22 via injecting with an adeno-associated virus was conducted to elucidate the role of IL-22 in *C. tyrobutyricum* attenuating colonic inflammation. The results showed that *C. tyrobutyricum* decreased the mRNA expression of IL-6 and IL-1β. *C. tyrobutyricum* enhanced the mRNA expression of IL-22 and the expression of MUC2 in the colon. The in vivo interference results showed that *C. tyrobutyricum* enhanced the mRNA expression of IL-6 and IL-1β while decreased the expression of MUC2 after knocking down IL-22. The flow cytometric analysis showed that *C. tyrobutyricum* decreased the proportions of macrophages, DCs, and mast cells and effectively regulated the proportion of Th17 cells, indicating that *C. tyrobutyricum* may stimulate the expression of IL-22 via regulating Th17 cells. Our study concluded that *C. tyrobutyricum* protected against LPS-induced colonic barrier dysfunction and inflammation via IL-22 signaling, suggesting that *C. tyrobutyricum* could be a potential probiotic in regulating colonic health.

## 1. Introduction

Inflammatory bowel disease (IBD), including Crohn’s disease and ulcerative colitis, is a chronic and incurable intestinal disease ranging from the old to the young. In 2015, over 1 million patients in the US and 2.5–3.0 million patients in Europe had IBD [1]. The incidence of IBD in western countries is considerably higher than that in some Asian countries like China and India. However, it is estimated that there will be more than 1.5 million IBD cases in China by 2025 [1]. IBD results from a complex interplay among host genetic, microbial, dietary, and environmental factors [2,3,4,5,6,7]. When IBD occurs, the harmful bacteria disseminate to the mucosal microenvironments, disrupting the homeostasis of the epithelial barrier, host flora, and immune cells. Thus, the interactions of intestinal microbiota, intestinal epithelial cells (IECs), and immune cells are not only important for the IBD but also vital for maintaining intestinal homeostasis.

Interleukin-22 (IL-22) was recently identified as one of the members of IL-10 family cytokines produced by Th17 cells, innate lymphoid cells (ILCs), NK and NKT cells, LTi cells, and non-lymphoid cells such as pulmonary macrophages, dendritic cells (DCs), and bone marrow-derived dendritic cells (BMDCs) [8,9]. The widespread effects of IL-22 in regeneration, host defense, and pathogenesis have been well described in various tissues, including the gut, lung, synovium, pancreas, liver, kidney, heart, thymus, and skin [10]. Most studies have shown that IL-22 is a mediator in intestinal epithelial cells and immune cell interactions. Secretion of IL-22 by immune cells induces proliferation of stem cells, thereby stimulating regeneration of damaged epithelial cells. However, the role of IL-22 in balancing gut microbiota, intestinal epithelial cells, and immune cells is still being elucidated.

Probiotics, defined as live bacteria, have been clinically used in alleviating gastrointestinal symptoms [11], fortifying the immune system [12], protecting against infectious diseases [13], and preventing metabolic diseases [14]. Although the mechanistic investigation of probiotics is limited by the cell-culture systems imitating the complex mucosa microenvironments, the beneficial effects of probiotics occur via multiple mechanisms, including improvement of epithelial barrier function, regulation of immunomodulation, suppression of pathogens/virus [15].

*C. tyrobutyricum* ATCC25755 (Ct) is a *Clostridium* spp. and high-producing butyric acid bacteria [16,17]. Our previous study showed that Ct protected against LPS-induced epithelial dysfunction in IPEC-J2 cells [17]. However, the function of Ct regulating intestinal immunity in vivo is still unknown. Thus, in this study, we first studied the role of Ct in regulating colonic inflammation. In vivo interference via injecting with the adeno-associated virus was conducted to elucidate the role of IL-22 in Ct attenuating LPS-induced colonic barrier dysfunction and inflammation.

## 2. Materials and Methods

### 2.1. Mice and Bacteria

Four-week-old C57BL/6 male mice were purchased from Shanghai SLAC Lab Animal Co., Ltd. (Shanghai, China). The mice experiments were fed at the Zhejiang University Laboratory Animal Center and conducted following the protocol approved by the Institutional Animal Care and Use Committee of Zhejiang University (Approval number: ZJU20200005). Ct was cultured in the clostridial growth medium (CGM) with controlled temperature (37 °C) and anaerobic environments. Ct was collected after centrifugation at 12,000 rpm for 5 min and suspended in PBS before gavage. Adeno-associated virus 2/2-m-shIL22 and AAV-shNC were purchased from Hanbio (Shanghai, China). 200 μL AAV-shIL22/AAV-shNC (negative control) with a concentration of 2.4 × 10^11^ vg/mL was injected intraperitoneally for two weeks. The sequences of AAV-shNC and AAV-shIL-22 were (5′ to 3′): 5′-TTCTCCGAACGTGTCACGTAA-3′ and 5′-GCTAAGGATCAGTGCTACCTGATGA-3′, respectively.

### 2.2. RT–qPCR

Total RNA of colonic samples was extracted using TRIzol reagent (Invitrogen, Carlsbad, CA, USA). cDNA was synthesized with MonScriptTM RTIII all-in-one mix with dsDNase (Monad, Wuhan, China) according to the manufacturer’s instructions. RT–qPCR was conducted on the CFX96TM real-time system (Bio-Rad) in triplicate with MonAmpTM SYBR Green qPCR Mix (Monad, Wuhan, China). Data were analyzed according to the 2^−ΔΔCt^ method and normalized to the expression of GAPDH. PCR primers are shown in Table 1.

### 2.3. Immunohistochemistry

The colonic samples were fixed in 4% PFA for 24 h and dehydrated in 30% sucrose for 48 h until sunk to the bottom. Colonic sectioning and immunohistochemistry were performed by Zhejiang Chinese Medical University. Briefly, the colonic sections were dewaxed with xylene, rehydrated through a graded series of ethanol solutions. After microwave treatment, the sections were blocked in 2.5% BSA and then incubated with MUC2 primary antibody (Proteintech, Wuhan, China) overnight followed by HRP-conjugated secondary antibodies (Abcam, Cambridge, UK) for 1 h. Images were scanned in the microscopy and visualized with NDP.View software (Hamamatsu, Beijing, China).

### 2.4. Colonic Lamina Propria Cell Isolation

The isolation of colonic lamina propria cells was conducted and improved following the protocol described by Scott et al. [18]. Briefly, the colon was dissected longitudinally and washed in PBS twice. The clean colonic sections were cut into 5-cm pieces and shaken in PBS containing 2% FBS, 10 mM HEPES, and 2 mM EDTA at 37 °C for 30 min for two cycles. The colonic pieces were digested in RPMI 1640 medium (10% FBS, 2 mM L-glutamine, 100 U/mL penicillin, 100 μg/mL streptomycin, 0.6 mg/mL collagenase VIII, and 150 μg/mL DNase) at 37 °C for 30 min. The completely digested tissues were then passed through 100 μm and 40 μm cell strainers. The intestinal lamina propria cells were obtained after centrifuging at 400× *g* for 10 min.

### 2.5. Flow Cytometry

The isolated intestinal lamina propria cells first were stain with Fixable Viability Dye 780 (FVS780) for 20 min and washed in PBS twice. Cells were stained with CD16/32 for 15 min to block the nonspecific binding to Fc receptors. The cells were then stained with fluorescence-labeled surface markers CD45-PerCP-Cy5.5, CD3e-BV510, CD4-PE-Cy7, CD25-BB515, CD127-BV421, CD117-BV421, FceRI-PE, CD11b-FITC, F4/80-AF647, and CD11c-PE-Cy7. These antibodies were purchased from B&D (San Jose, CA, USA). For intracellular transcription factor staining, cells were fixed and permeabilized for 1 h, followed by staining with intracellular markers including Foxp3-APC (B&D, San Jose, CA, USA), T-bet-AF647 (B&D, USA), GATA3-BV421 (B&D, San Jose, CA, USA), and RORγt-PE (eBioscience, Waltham, MA, USA) for another 1 h. All flow cytometric experiments were performed using FACS Verse (BD Biosciences). Cells were first gated on FSC-A versus SSC-A, single cells (FSC-H versus FSC-A), and CD45^+^FVS780^−^ cells. The macrophages (CD11b^+^F4-80^+^), mast cells (CD117^+^FceRI^+^), and DCs (CD11c^+^CD11b^−^) were gated out from CD45^+^FVS780^−^CD3e^−^ cells. Th1 cells (T-bet^+^) and Th2 cells (GATA-3^+^) were gated out from CD45^+^FVS780^−^CD3e^+^CD4^+^ cells. T cells (CD3e^+^CD4^+^) were gated out from CD45^+^FVS780^−^ cells. ILC3s (CD45^+^CD3e^−^CD127^+^RORγt^+^) were gated out from CD45^+^FVS780^−^CD3e^−^ cells. Tregs (CD25^+^Foxp3^+^) and Th17 cells (CD3e^+^RORγt^+^) were gated out from CD45^+^FVS780^−^CD3e^+^CD4^+^ cells.

### 2.6. Statistical Analysis

All statistics were analyzed with Prism 8.0 software. A two-tail unpaired *t*-test was employed between the two groups. All data are presented as mean ± SEM, and *p* < 0.05 were considered significant. Flow cytometric data were analyzed with FlowJo 10.0 software. The average optical density (AOD) of immunohistochemistry was analyzed with ImageJ software.

## 3. Results

### 3.1. Ct Attenuated Colonic Inflammation in Mice

**Figure 1:**
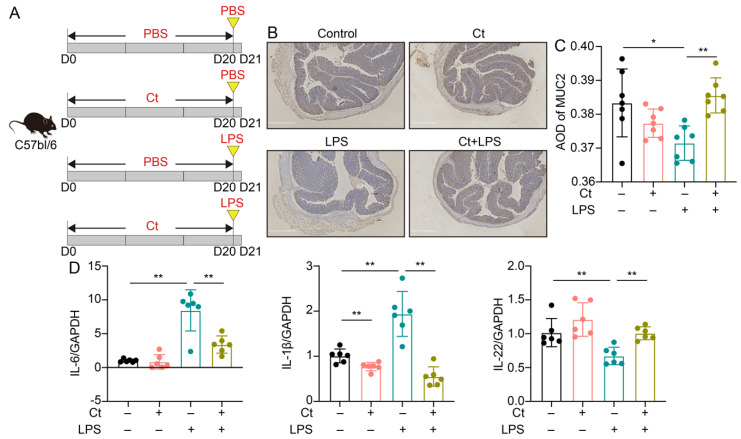
*C. tyrobutyricum* ATCC25755 (Ct) efficiently attenuated colonic inflammation in mice. (**A**) Schematic for the feeding experiment. C57BL/6 mice were treated with phosphate buffer saline (PBS) or 10^8^ CFU/mL Ct by gavage every day for 20 days followed by intraperitoneal injection with PBS or lipopolysaccharide (LPS) (10 mg/kg, Body Wight, BW) at day 20 (D20), the samples were collected after 24 h (Control, *n* = 10; Ct, *n* = 10; LPS, *n* = 12; Ct + LPS, *n* = 12); (**B**) immunohistochemistry images of colonic MUC2. Scale bars: 250 μm; (**C**) average optical density (AOD) of MUC2; (**D**) relative mRNA expression of IL-6, IL-1β, and IL-22 in the colon (*n* = 6). Data presented as mean ± SEM. * *p* < 0.05, ** *p* < 0.01 by two-way unpaired *t*-tests.

In this study, we used LPS to induce intestinal inflammation and dysfunction. To investigate the effects of Ct on attenuating colonic inflammation and barrier dysfunction in mice, we administered 10^8^ CFU/mL Ct by gavage for 20 days, followed by intraperitoneal injection with LPS (10 mg/kg BW) for 24 h (Figure 1A). We first evaluated the effects of Ct on the expression of MUC2 using immunohistochemistry. No significant differences were showed between mice treated with PBS (control) and Ct alone (*p* = 0.1710, Figure 1B,C). Compared with mice injected with LPS alone, the expression of MUC2 was increased in mice pretreated with Ct (*p* < 0.01, Figure 1B,C).

No differences could be observed in mRNA expression of IL-6 and IL-22 between mice treated with PBS and Ct, although Ct relatively decreased the mRNA expression of IL-1β compared with the control (*p* < 0.01, Figure 1D). These results showed that supplementation of Ct was not harmful to intestinal immunity. Under the inflammatory condition, compared with mice injected with LPS, the mRNA expression of IL-1β and IL-6 was decreased while the mRNA expression of IL-22 was significantly increased in mice pretreated with Ct (*p* < 0.01, Figure 1D). These results indicated that Ct efficiently attenuated LPS-induced colonic inflammation in mice.

### 3.2. Effects of Ct on Immune Cells in Mice

To further investigate the role of Ct in regulating intestinal immunity, we isolated the immune cells in lamina propria and analyzed the proportions of immune cells in the innate and adaptive immune systems using flow cytometry after the feeding trial.

#### 3.2.1. Effects of Ct on Macrophages, Mast Cells, and DCs in Mice

**Figure 2:**
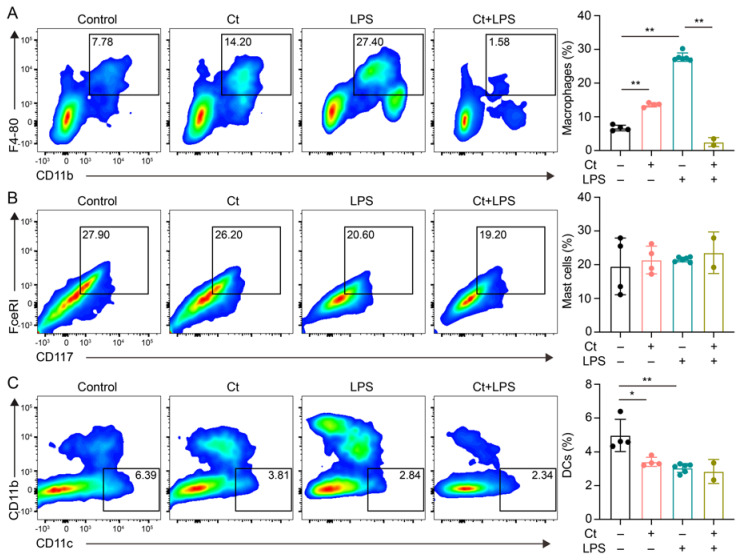
Effects of Ct on proportions of macrophages, mast cells, and dendritic cells (DCs) in mice. (**A**) Proportion of macrophages; (**B**) proportion of mast cells; (**c**) proportion of DCs. Each dot presented one mouse. Data presented as mean ± SEM. * *p* < 0.05, ** *p* < 0.01 by two-way unpaired *t*-tests.

We first analyzed the proportions of macrophages, mast cells, and DCs. As shown in Figure 2, compared with mice treated with PBS (control), supplementation of Ct alone enhanced the proportion of macrophages (*p* < 0.01, Figure 2A) and decreased the proportion of DCs (*p* < 0.01, Figure 2C). However, under the inflammatory condition, the proportion of macrophages was dramatically decreased in mice pretreated with Ct, compared with mice injected with LPS (*p* < 0.01, Figure 2A). No significant differences were observed in the proportion of DCs between mice treated with LPS or mice pretreated with Ct (*p* = 0.5258, Figure 2C). Moreover, there were no significant differences in the proportion of mast cells among the treatments.

#### 3.2.2. Effects of Ct on T Cells and Its Cell Subsets in Mice

**Figure 3:**
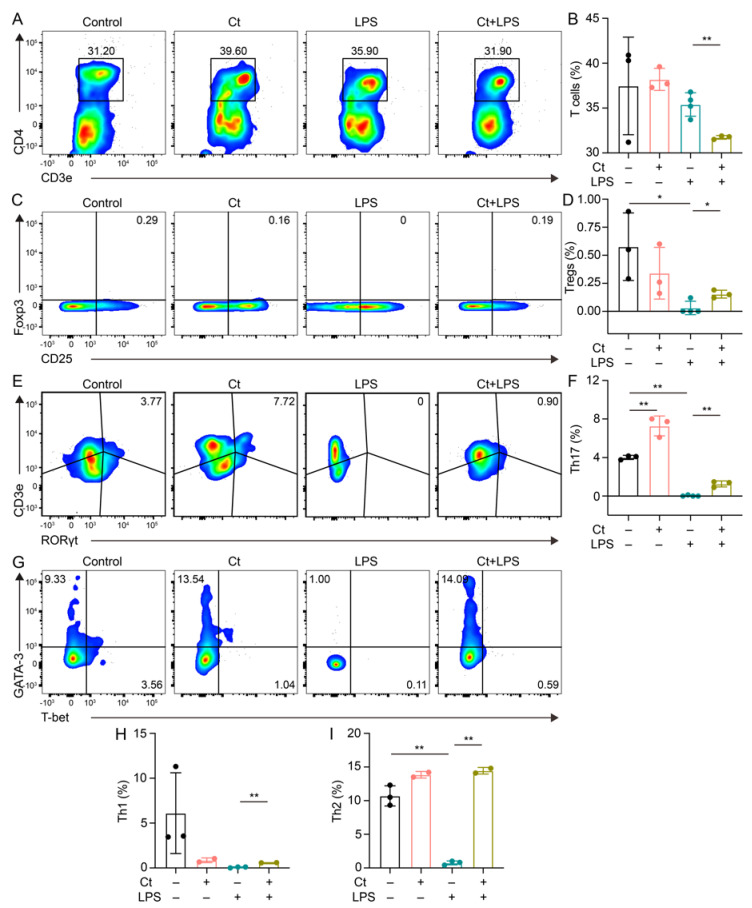
Effects of Ct on the proportion of T cells and its subsets in mice. (**A**,**B**) Proportion of T cells; (**C**,**D**) proportion of Tregs; (**E**,**F**) proportion of Th17 cells; (**G**,**I**) proportions of Th1 and Th2 cells. Each dot presented one mouse. Data presented as mean ± SEM. * *p* < 0.05, ** *p* < 0.01 by two-way unpaired *t*-tests.

We next evaluated the proportion of T cells and T cell subsets in the colon. Compared with Control, mice supplemented with Ct did not affect the proportion of T cells (*p* = 0.8309, Figure 3A,B). However, compared with mice injected with LPS alone, the proportion of T cells was decreased in mice pretreated with Ct in the inflamed status (*p* < 0.01, Figure 3A,B).

T cell subsets were then analyzed in this study. We found that Ct enhanced the proportion of Th17 cells (*p* < 0.01, Figure 3E,F), but had no effects on other T cell subsets including Tregs, Th1, and Th2 cells (*p* > 0.05, Figure 3C,D,G–I), compared with the control. Under the inflammatory condition, the proportions of both Tregs and Th17 cells were increased in the mice pretreated with Ct under inflamed condition, compared with mice injected with LPS alone (*p* < 0.05, *p* < 0.01, respectively, Figure 3C–F. Moreover, compared with mice injected with LPS, the proportions of both Th1 and Th2 were enhanced in mice pretreated with Ct (*p* < 0.01, Figure 3G–I).

#### 3.2.3. Effects of Ct on ILC3s in Mice

**Figure 4:**
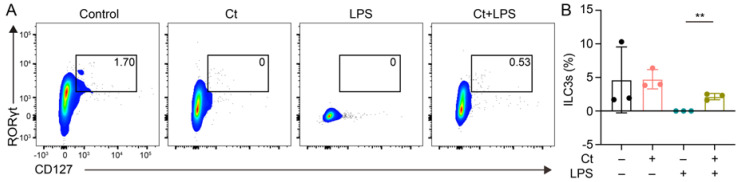
Effects of Ct on proportion of ILC3s in mice. Each dot presented one mouse. Data presented as mean ± SEM. ** *p* < 0.01 by two-way unpaired *t*-tests.

The proportion of ILC3s was analyzed in this study. As shown in Figure 4, the supplementation of Ct had no effect on ILC3s proportion, compared with mice treated with PBS (*p* = 0.9754). However, the proportion of ILC3s was enhanced in mice pretreated with Ct, compared with mice injected with LPS (*p* < 0.01).

### 3.3. Ct Protected against Colonic Inflammation and Barrier Dysfunction via IL-22

**Figure 5:**
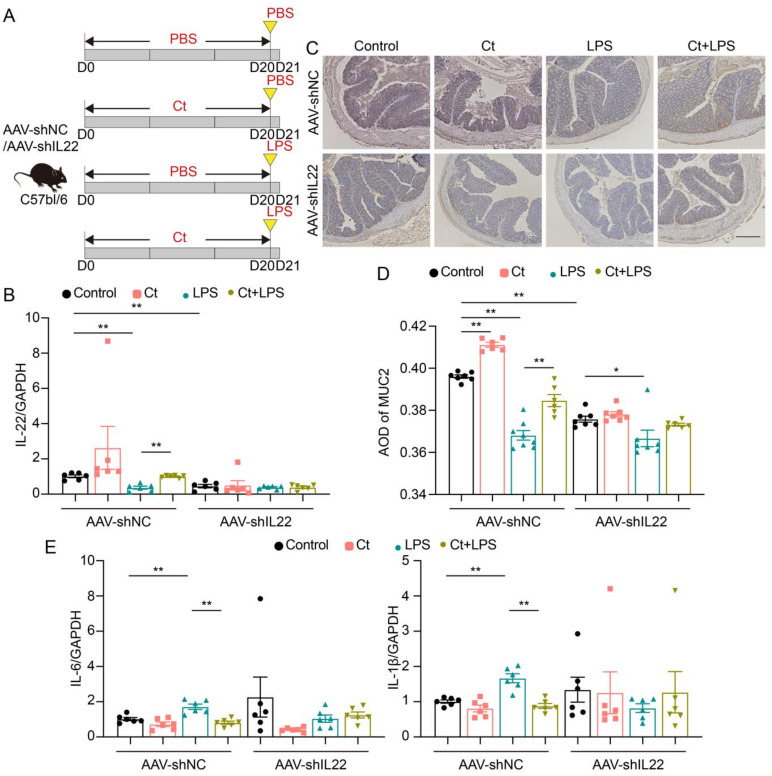
Ct protected against colonic barrier dysfunction and inflammation via IL-22 in mice. (**A**) Schematic for the feeding experiment. C57BL/6 mice were first intraperitoneal injection with AAV-shNC or AAV-shIL22 for 2 weeks, followed by treatments with PBS or 10^8^ CFU/mL Ct by gavage every day for 20 days followed by intraperitoneal injection with PBS or LPS (10 mg/kg BW) at day 20 (D20), the samples were collected after 24 h (control, *n* = 19 in AAV-shNC, *n* = 17 in AAV-shIL22), Ct (*n* = 14 in AAV-shNC, *n* = 12 in AAV-shIL22), LPS (*n* = 20 in AAV-shNC, *n* = 18 in AAV-shIL22), and Ct + LPS (*n* = 17 in AAV-shNC, *n* = 12 in AAV-shIL22); (**B**) relative mRNA expression of IL-22 (*n* = 6); (**C**) immunohistochemistry images of colonic MUC2. Scale bars: 250 μm; (**D**) average optical density (AOD) of MUC2; (**E**) relative mRNA expression of IL-6 and IL-1β (*n* = 6). Data presented as mean ± SEM. * *p* < 0.05, ** *p* < 0.01 by two-way unpaired *t*-tests.

As shown in Figure 1D, the mRNA expression of IL-22 was increased in the mice pretreated with Ct. We then hypothesized whether Ct protected against intestinal inflammation via IL-22 signaling. To fully understand the role of IL-22 in Ct regulating intestinal health, IL-22 was knocked down in vivo via injecting with the adeno-associated virus for two weeks, and the feeding experiment with Ct gavage followed by LPS was conducted (Figure 5A).

The mRNA expression of IL-22 was decreased in mice injected with AAV-shIL22, compared with mice injected with AAV-shNC (*p* < 0.01, Figure 5B), indicating that IL-22 was successfully knocked down in mice after injecting with AAV-shIL22 for two weeks. In the mice injected with AAV-shNC, the mRNA expression of IL-22 was enhanced in mice pretreated with Ct, compared with mice injected with LPS (*p* < 0.01, Figure 5B). However, after knocking down IL-22 in vivo, no significant differences could be observed in the mRNA expression of IL-22 among the treatments (Figure 5B).

We then analyzed the expression of MUC2 using immunohistochemistry (Figure 5C,D). In mice injected with AAV-shNC, compared with the control, the expression of MUC2 was enhanced in mice treated with Ct alone (*p* < 0.01), however, compared with mice injected with LPS, the expression of MUC2 was increased in mice pretreated with Ct (*p* < 0.01). No significant differences could be observed between mice treated with LPS and pretreated with Ct after knocking down IL-22 (*p* > 0.05).

As shown in Figure 5E, in mice without knocking down IL-22, Ct dramatically decreased the mRNA expression of IL-1β and IL-6, compared with mice injected with LPS (*p* < 0.01). However, after knocking down IL-22, no differences were observed among treatments. Altogether, these results suggested that Ct protected against colonic barrier dysfunction and inflammation depending on IL-22.

### 3.4. Effects of Ct on Immune Cells in Mice Knocking down IL-22

To elucidate the role of IL-22 in Ct regulating the colonic immune cells, we isolated the lamina propria immune cells after the in vivo interference feeding trial.

#### 3.4.1. Effects of Ct on Macrophages, Mast Cells, and DCs in Mice Knocking down IL-22

**Figure 6:**
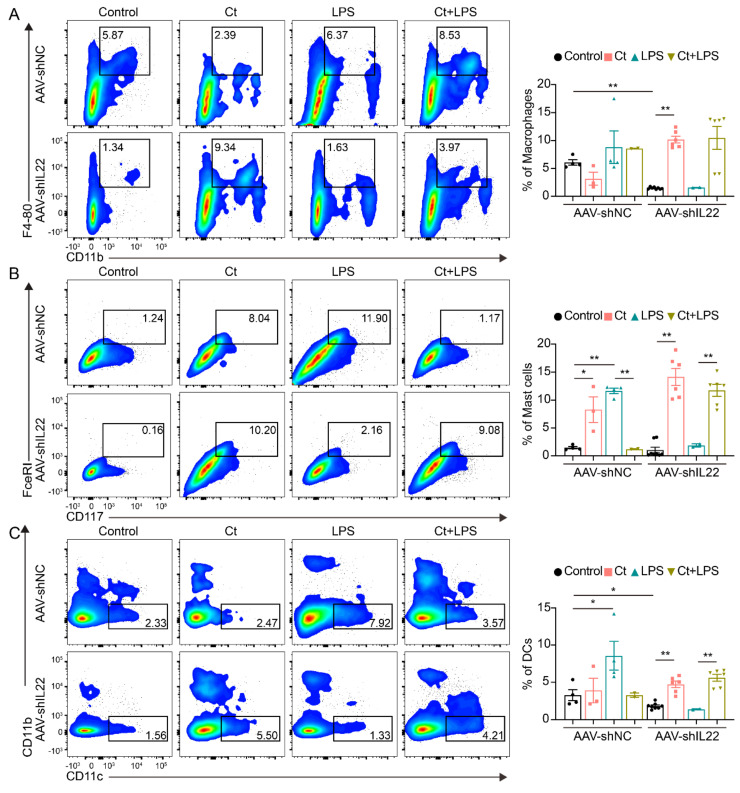
Effects of Ct on proportions of macrophages, mast cells, and dendritic cells (DCs) in mice knocking down IL-22. (**A**) Proportion of macrophages; (**B**) proportion of Mast cells; (**C**) proportion of DCs. Each dot presented one mouse. Data presented as mean ± SEM. * *p* < 0.05, ** *p* < 0.01 by two-way unpaired *t*-tests.

No significant differences were observed in the proportion of macrophages in AAV-shNC-treated mice among the treatments. However, after knocking down IL-22, compared with the control, the proportion of macrophages was significantly enhanced in mice treated with Ct alone (*p* < 0.01, Figure 6A). The proportion of macrophages was relatively increased in mice pretreated with Ct, although no significant differences could be observed compared with mice treated with LPS alone after knocking down IL-22 (Figure 6A). In the mice injected with AAV-shNC, Ct decreased the proportion of mast cells but had no effects on the proportion of DCs, compared with mice treated with LPS (Figure 6B,C). Compared with mice injected with LPS, the proportions of mast cells and DCs were significantly enhanced in mice pretreated with Ct after knocking down IL-22 (*p* < 0.01, Figure 6B,C).

#### 3.4.2. Effects of Ct on T Cells, Tregs, and Th17 Cells in Mice Knocking down IL-22

**Figure 7:**
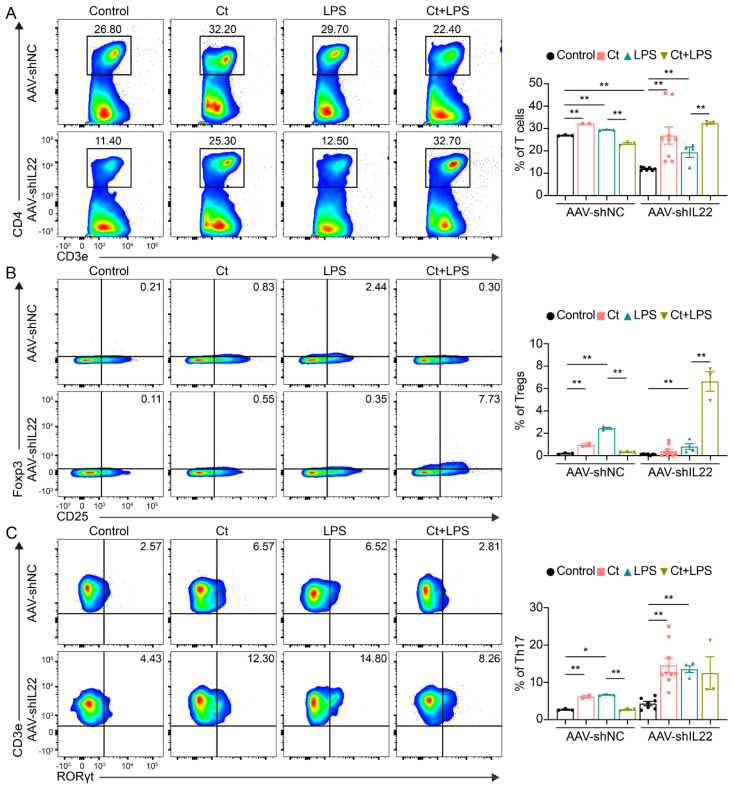
Effects of Ct on proportions of T cells, Tregs, and Th17 cells in mice knocking down IL-22. (**A**) Proportion of T cells; (**B**) proportion of Tregs; (**C**) proportion of Th17 cells. Each dot presented one mouse. Data presented as mean ± SEM. * *p* < 0.05, ** *p* < 0.01 by two-way unpaired *t*-tests.

As shown in Figure 7, in AAV-shNC-treated mice, supplementation of Ct alone increased the proportions of T cells, Tregs, and Th17 cells compared with mice treated with PBS (control) (*p* < 0.01). Moreover, the proportions of T cells, Tregs, and Th17 cells were decreased in mice pretreated with Ct compared with mice treated with LPS (*p* < 0.01). After knocking down IL-22, compared with the AAV-shIL22-treated mice injected with LPS, the proportions of T cells and Tregs were significantly increased in mice pretreated with Ct (*p* < 0.01). However, no significant differences were observed in the proportion of Th17 cells between AAV-shIL22-treated mice injected with LPS alone and pre-supplemented with Ct.

#### 3.4.3. Effects of Ct on Th1 and Th2 Cells in Mice Knocking down IL-22

**Figure 8:**
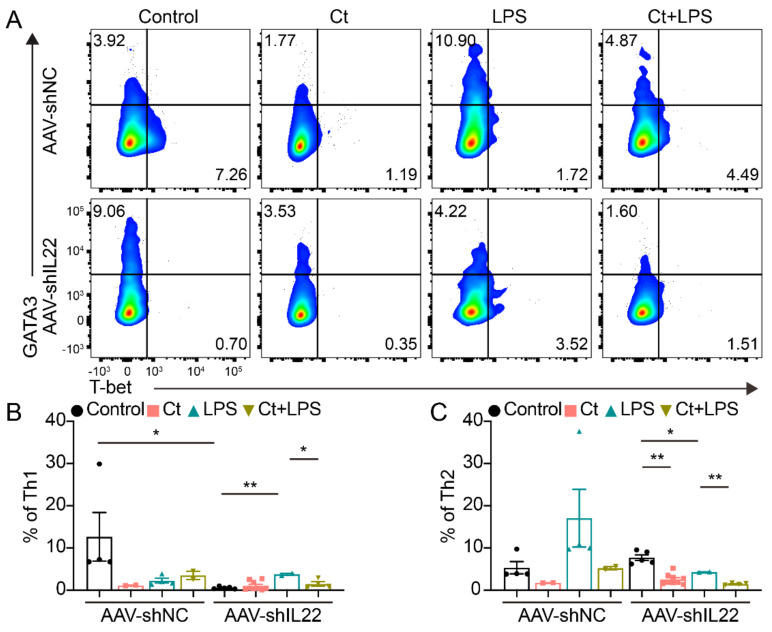
Effects of Ct on regulating colonic Th1 and Th2 cells in mice knocking down IL-22. (**A**) Flow cytometric dots of Th1 and Th2 cells; (**B**) proportion of Th1 cells; (**C**) proportion of Th2 cells. Each dot presented one mouse. Data were presented as mean ± SEM. * *p* < 0.05, ** *p* < 0.01 by two-way unpaired *t*-tests.

As shown in Figure 8, no significant differences could be observed in mice injected with AAV-shNC among the treatments. However, after knocking down IL-22 in vivo, the proportion of Th2 was decreased in mice treated with Ct alone compared with the control (*p* < 0.01). In addition, the proportions of Th1 and Th2 were decreased in mice pretreated with Ct, compared with mice injected with LPS (*p* < 0.05, *p* < 0.01, respectively).

#### 3.4.4. Effects of Ct on ILCs in Mice Knocking down IL-22

**Figure 9:**
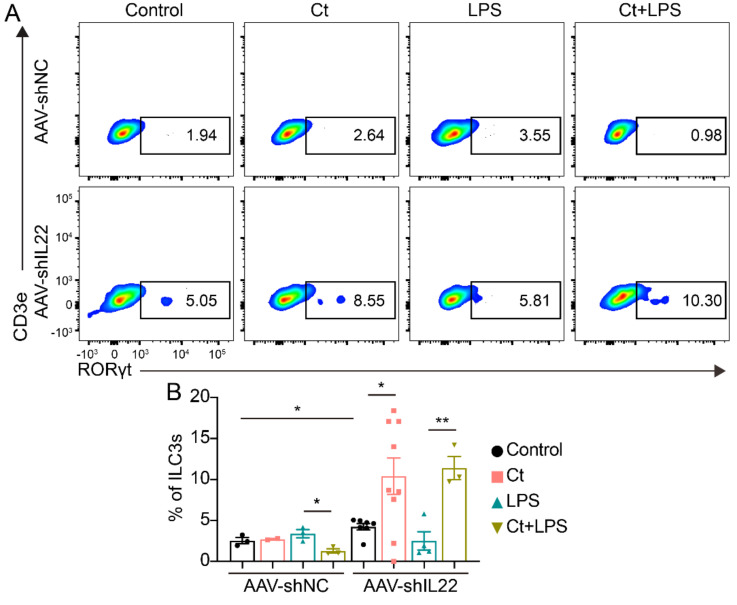
Effects of Ct on regulating colonic ILC3s in mice knocking down IL-22. Each dot presented one mouse. Data presented as mean ± SEM. * *p* < 0.05, ** *p* < 0.01 by two-way unpaired *t*-tests.

In AAV-shNC-treated mice, the proportion of ILC3 was decreased in mice pretreated with Ct compared with this in mice injected with LPS (*p* < 0.05, Figure 9). After knocking down IL-22, Ct enhanced the proportion of ILC3, compared with the control (*p* < 0.05). Moreover, the proportion of ILC3s was enhanced in mice pre-supplemented with Ct, compared with mice treated with LPS (*p* < 0.01, Figure 9).

## 4. Discussion

The application of probiotics is controversial due to some unsolved problems, such as safety, colonization, and effects on the indigenous microbiome. However, probiotics have been clinically suggested to be effective in preventing intestinal diseases like acute gastroenteritis, bacteria-associated diarrhea, irritable bowel syndrome (IBS), and IBD [19,20]. Ct, a Gram-positive anaerobic bacterium and our previous study has shown that Ct protects the intestinal epithelial barrier in IPEC-J2 cells [17]. The genomic analysis reveals that Ct contains multiple genes encoding biological enzymes and does not possess harmful genes, indicating that Ct is a promising microbe for human health and industrial applications [21]. Consistent with the analysis of the genome sequence of Ct, we first verified that Ct protected against colonic inflammation in vivo (shown in Figure 1).

The intestinal barrier is the first line to protect the gastrointestinal tract, and the mucus layer has a vital role in preventing chemical and biological disruptions and maintaining intestinal homeostasis [22,23]. The mucin not only protects epithelial cells from dehydration and mechanical stress, but it also has the ability to remove debris and bacteria via the intestinal flow [22,24,25]. MUC2 is the main component of the intestinal mucus in both the small and large intestine. In this study, we found that the expression of MUC2 was enhanced in mice pretreated with Ct, indicating that Ct efficiently protected against epithelial barrier function under the inflammatory stimulation (shown in Figure 1). These findings suggested that Ct is a promising probiotic and has potential applications in gastrointestinal diseases like IBD.

Interleukin-22 (IL-22), originally named IL-10 related to T cell-derived inducible factor (IL-TIF), was discovered by Gurney’s group and Renauld’s group in 2000 [26,27]. The widespread effects of IL-22 in regeneration, host defense, and pathology have been described in numerous tissues, including lung, synovium, pancreas, liver, kidney, heart, thymus, skin, and gut [10]. However, the function of IL-22 in barrier organs is controversial, especially in the intestine. Earlier studies have revealed that IL-22 is highly expressed in chronic inflammatory conditions including psoriasis, IBD, and rheumatoid arthritis and induces expression of proinflammatory molecules like IL-6, IL-1, GM-CSF, and LPS-binding protein [28,29,30,31]. A recent study shows that TGF-β signaling and strong TCR stimulation, coupled with AHR ligands, promote IL-22 production by Th17 cells and cause colitis-associated colon cancer [32]. On the contrary, IL-22 is regarded as a predominantly beneficial cytokine if it is controlled properly. A well-known mechanism for IL-22 repairs tissue-damages and protects against pathogens is by promoting the regeneration of epithelial cells and production of antimicrobial molecules like Reg3β, β-defensin, and S100 [10,33,34]. In this study, we found that Ct enhanced the mRNA expression of IL-22 and prevented inflammation in mice without injecting with an adeno-associated virus, indicating that the enhanced IL-22 by Ct may contribute to the protection against colonic dysfunction (shown in Figure 1). These results guided us to hypothesized whether Ct protected colonic homeostasis depending on IL-22. Thus, we knocked down IL-22 in vivo via injecting adeno-associated virus labeled with GFP for two weeks. Our unpublished data showed that the adeno-associated virus specifically targeted the ileal and colonic epithelial cells, indicating that the IL-22 was successfully knocked down in the colonic epithelial cells. In this study, after knocking down IL-22, no significant differences could be observed in mRNA expression of inflammatory cytokines and expression of MUC2 between mice injected with LPS or pretreated with Ct (shown in Figure 5). Strangely, the mRNA expression of inflammatory cytokines was decreased in response to LPS after knocking down IL-22. Previous studies have shown that IL-22 administration upregulates LPS-binding protein blood level, reaching concentrations known to neutralize LPS in IBD patients [10,30]. However, whether LPS induces colonic inflammation depending on IL-22 needs further investigation considering the complex gut microenvironment. Altogether, our findings showed that Ct protected colonic health via IL-22. Moreover, our study verified the protective function of IL-22 in intestinal inflammation, which was consistent with the results of others [10,33,34].

The epithelial cells receive signals from diet and microbiota and orchestrate the communication between the microbiota in the lumen and immune cells in the lamina propria. The intestinal inflammation cannot only result in the alteration of gut microbiota (known as dysbiosis), a typical characteristic of IBD [35], it also affects the function of immune cells in the lamina propria. According to the historical subdivision of mononuclear phagocytes, the intestinal mononuclear phagocytes are subdivided into dendritic cells (DCs) and macrophages, responding to microbes and other potential stimuli [36,37]. When tissue damage occurs, the innate immune system detects the damaged-associated molecular patterns and pathogen-associated molecular patterns and induces neutrophils infiltration, which in turn recruit inflammatory monocytes to stimulate response to the inflammation [38,39]. Moreover, DCs are defined as antigen presentation cells and orchestrate innate and adaptive immune responses [40]. The activation of DCs induced by infectious agents and inflammatory products induces the production of cytokines, enhances the expression of genes in the MHC-II process, and activates T cells [41]. In the innate immune system, the role of mast cells is in an antigen-independent and adaptive immune-independent manner. In intestinal disorders like IBD, the proportion of mast cells is higher than in the normal intestines, and the enhanced levels of histamine produced by mast cells as well as the expression of histamine receptors exacerbate the immune cell recruitment and inflammation [42,43,44]. In this study, we found that Ct decreased the proportions of macrophages, DCs, mast cells, and T cells in AAV-shNC-treated mice, although some of the results were not completely consistent in mice without injecting with an adeno-associated virus. After knocking down IL-22 in vivo, the proportions of these immune cells were dramatically increased. Taken together, our study suggested that Ct may protect colonic health via inhibiting the inflammatory function of mononuclear phagocytes and activation of T cells and the protective function of Ct was dependent on IL-22.

*C. butyricum* is clinically used as a probiotic in diarrhea, colitis, and IBD in both human and production animals. *Clostridium* species have been shown to suppress colitis via promoting the subsequent accumulation of IL-10-producing intestinal Treg cells and providing bacterial antigens and a TGF-β-rich environment to help the differentiation of Treg cells in mice [45,46]. Moreover, its metabolites like butyrate enhanced histone H3 acetylation in the promoter of Foxp3 locus leading to the differentiation of iTreg cells [47]. In the innate immune system, *C. butyricum* directly triggers IL-10 production by intestinal macrophages in inflamed mucosa, preventing colitis [48]. A recent study also shows that *C. butyricum* promotes iTreg cell generation through induction of TGF-β from DCs [49]. In our study, we observed that Ct enhanced the proportion of Tregs in the non-AAV-treated mice, which was consistent with the changes of Tregs in mice treated with *C. butyricum* [45,46]. However, the results were inconsistent with these in AAV-shNC-treated mice. Similarly, although Th1 and Th2 polarization were important in mucosal immunity, we found that the proportions of Th1 and Th2 were controversial in non-AAV-treated mice and AAV-shNC-treated mice. Thus, whether Ct protected against colonic inflammation via Tregs or Th1/Th2 cells needed further study.

Generally, IL-22 is mainly produced by Th17 cells and ILC3s. IL-22 has been regarded as a mediator in IECs-immune interactions. Numerous studies have shown that IL-22 secreted by ILC3s and Th17 cells promotes intestinal-stem-cell-mediated epithelial regeneration, protects intestinal stem cells against genotoxic stress, and regulates intestinal epithelial permeability [10,32,33,34,50,51]. Although tryptophan metabolites from microbiota engage aryl hydrocarbon receptor (AhR) and balance mucosal reactivity via IL-22 and highly adaptive lactobacilli produces AhR ligand-indole-3-aldehyde contributing IL-22-dependent balanced mucosal response and providing colonization resistance to fungus *Candida albicans* and mucosal protection from inflammation [50]. In our study, we found that Ct enhanced the proportion of ILC3 in both non-AAV-treated mice and IL-22 knocked down mice. However, the results in mice without injecting with an adeno-associated virus and AAV-shNC-treated mice were inconsistent. Thus, whether Ct could regulate ILC3s needed to be clarified. According to the flow cytometric analysis, we did not find significant differences between mice injected with LPS or pretreated with Ct after knocking down IL-22, although the proportions of Th17 cells in non-AAV-treated mice and AAV-shNC-treated mice were opposite. These results indicated that Ct might enhance IL-22 expression via regulating Th17 cells in the colon, thereby protecting against colonic inflammation.

## 5. Conclusions

In conclusion, Ct inhibited the inflammatory function of immune cells in the innate immune system and may stimulate the expression of IL-22 via regulating Th17 cells. More important, Ct was efficient in protecting against colonic epithelial dysfunction and inflammation via IL-22 signaling. 

## Figures and Tables

**Table 1:** Gene-specific primers used in RT–qPCR experiments.

Genes	Forward Primer (5′-3′)	Reverse Primer (5′-3′)
GAPDH	AAGAAGGTGGTGAAGCAGGCATC	CGGCATCGAAGGTGGAAGAGTG
IL-6	ACTTCCATCCAGTTGCCTTCTTGG	TTAAGCCTCCGACTTGTGAAGTGG
IL-1β	TCGCAGCAGCACATCAACAAGAG	TGCTCATGTCCTCATCCTGGAAGG
IL-22	TCCAACTTCCAGCAGCCATACATC	GCACTGATCCTTAGCACTGACTCC

## Data Availability

All data supporting the findings of this study are available within the figures. Raw data are available on request from the corresponding author.
